# Ocular Biometry Percentile Curves and Their Relation to Myopia Development in Indian Children

**DOI:** 10.3390/jcm13102867

**Published:** 2024-05-13

**Authors:** Aparna Gopalakrishnan, Viswanathan Sivaraman, Jameel Rizwana Hussaindeen, Meenakshi Swaminathan, Alex Gentle, James A. Armitage, Simon Backhouse

**Affiliations:** 1 Myopia Clinic, Sankara Nethralaya, Unit of Medical Research Foundation, Chennai 600006, India; viswa0308@gmail.com (V.S.); rizwanaopto@gmail.com (J.R.H.); meenakshiswaminathan@gmail.com (M.S.); 2 School of Medicine, Faculty of Health, Deakin University, Waurn Ponds, VIC 3216, Australia; alex.gentle@deakin.edu.au (A.G.); j.armitage@deakin.edu.au (J.A.A.); simon.backhouse@deakin.edu.au (S.B.); 3 R&D, EssilorLuxottica, Singapore 339338, Singapore

**Keywords:** myopia, axial length, AL/CR ratio, growth curves, percentiles, India

## Abstract

**Background:** The aim of the present study was to provide ocular biometry percentile values for Indian children between the ages of 6 and 12 and to validate the usefulness of centiles in predicting myopia development. **Methods:** The study was part of a longitudinal study—the Sankara Nethralaya Tamil Nadu Essilor Myopia Study (STEM), where objective refraction and ocular biometry were measured for children studying in grades 1, 4, and 6 at baseline (2019–2020). These data were used to generate ocular biometry percentile curves (both for axial length (AL) and AL/corneal curvature (AL/CR) ratios). The usefulness of percentile values in predicting myopia development was estimated from follow-up data (2022). **Results:** The total number of children in the three grades at baseline was 4514 (age range 6 to 12). Boys represented 54% (n = 2442) of the overall sample. The prevalence of myopia at baseline was 11.7% (95% CI from 10.8 to 12.7%) in these three grades. Both the AL and AL/CR ratio centiles showed a linear trend with an increase in AL and AL/CR with increasing grades (*p* < 0.001) for all percentiles (2, 5, 10, 25, 50, 75, 90, 95, 98, and 99) when stratified by sex. In the follow-up data (n = 377), the 75th and 50th percentiles of the AL/CR ratio had an area under the curve (AUC) of 0.79 and 0.72 to predict myopia onset for grade 4 and 6 children at baseline. Combining baseline AL with the centile shift in follow-up as a predictor increased the AUC to 0.83. **Conclusions:** The present study has provided centile values specific for Indian children between the ages of 6 and 12 to monitor and intervene where children are at a higher risk of myopia development.

## 1. Background

Myopia prevalence has increased globally, and in India, there has been a substantial increase in this prevalence recently [1,2] compared with the preceding decades [3,4]. Indeed, a recent study predicted that myopia prevalence will reach 50% in India by the year 2050 [5]. 

For these reasons, it is important to identify the risk of myopia onset among children at the earliest feasible stage to implement control measures in a timely manner. One of the key factors in myopia development is excessive ocular growth, in particular axial length and the ratio of axial length to corneal curvature. 

The refractive state of the eye at birth is, in most cases, hyperopic, and the eye then undergoes rapid change, through an increase in axial length and a reduction of corneal and lens power, to reach the state of emmetropia by the age of 6 years [6]. When there is a lack of coordination in the rate of change of these optical component parameters or when there is excessive axial length growth, refraction tends towards myopia. A study by Mutti et al., in myopic children between the ages of 6 and 12 years, reported that the rate of myopia progression increased until the age of 11, followed by a period of decelerated change and stabilization by age 15, with an ethnic dependency on the age of stabilization [7]. Furthermore, children with earlier-onset myopia had a faster rate of progression with a higher myopic refractive error at the time of stabilization [7]. On the other hand, a study in a European population has shown that emmetropic eyes continue to grow until the age of 18 years, though the rate of change in axial length decreases with age (0.17 mm at 6 to 0.02 mm at age 11 and above), and those with a higher axial length at baseline become myopic [8]. Given the correlation between optical components of the eye and refraction, especially at a younger age, monitoring the rate of change in axial length could therefore help in identifying children at risk of developing myopia. 

Growth percentile charts for height and weight have been commonly used in pediatric medicine for decades, and, in recent years, percentile charts have been developed for monitoring ocular growth to identify children at risk of myopia development [9,10,11,12,13]. Akin to height and weight charts needing to be developed for different ethnic populations, there is a need for the development of population-specific ocular biometric growth charts. Indeed, the extensive data describing ocular growth in Caucasian and ethnic Chinese populations may not hold true for Indian populations. 

The Sankara Nethralaya Tamil Nadu Essilor (STEM) study is an ongoing longitudinal study of approximately 14,000 children aimed at understanding the prevalence, incidence, and risk factors for myopia onset and progression among schoolchildren in South India [1,14]. As part of the STEM study, ocular biometry parameters were measured in a subset of children studying in grades 1, 4, and 6 (between the age ranges of 6 and 12). 

The aim of the present study, therefore, was to investigate the percentile charts for axial length and axial length to corneal curvature (AL/CR) ratio, developed from the cross-sectional baseline measurements of the STEM study, and to validate the usefulness of percentile measurements in identifying the risk of myopia development among South Indian children from longitudinal data. 

## 2. Methodology

The STEM study was approved by the Institutional Review Board and Ethics Committee of the Vision Research Foundation, Sankara Nethralaya (Study 730-2018-P, date of approval: 20 December 2018) and by the Deakin University Human Research Ethics Committee (DUHREC 2020–213, date of approval: 12 October 2020). The study followed the tenets of the Declaration of Helsinki. Written informed consents were obtained from the children’s parents before the school screening process commenced. A detailed description of the methods used for the baseline cross-sectional study has been published previously [1]. Briefly, children from a total of eleven schools in and around two districts of Tamil Nadu in South India underwent ocular assessment that involved visual acuity measurement, objective and subjective refraction measurements, binocular vision measurements, and ocular biometry measurements. Objective refraction was measured using an open-field auto-refractometer (Grand Seiko WAM 5500, Grand Seiko Co., Ltd., Hiroshima, Japan). The use of an open-field auto-refractometer ensured that accommodation was relaxed for distance viewing, potentially eliminating pseudomyopia due to excess accommodation. 

Subjective refraction was performed on children who had correction for existing refractive error and for those who failed visual acuity screening (acuity < 6/9.5). Children in grades one, four, and six also underwent ocular biometry measurements using an optical biometer (IOL Master version 5.4, Carl Zeiss, Oberkochen, Germany). Five readings of corneal curvature (CR), axial length (AL), and anterior chamber depth (ACD) were taken, and the average of the readings was considered the definitive measurement. The IOL Master was used as this is the gold standard biometer that has been extensively employed in previous epidemiological studies [15,16] and was previously validated for school screening data collection in South India [17]. Myopia was defined as a non-cycloplegic open-field autorefraction measurement of ≤−0.75 D [18]. Anterior chamber depth has been shown to have only a minor relationship with refractive error in Indian populations [19,20] and was not analyzed in the present study, although ACD results for the entire STEM study cohort have been published previously [1]. Binocular vision assessments included accommodative measurements, near-point of convergence, and phoria measurements. Children with poor visual acuity, despite subjective refraction, strabismus, amblyopia, binocular vision anomalies, and other ocular problems, were referred for further ophthalmic examination and were excluded from the study [1]. 

### 2.1. Data for Validation

For validation, children who were in grades 1, 4, and 6 in the first three schools visited during the baseline data collection were followed up after 3 years (mean follow-up: 35 months; range: from 33 to 39 months) in the year 2022. A total of 414 children (grade 1, n = 90; grade 4, n = 159; grade 6, n = 165) in the three grades who had complete refraction and ocular biometry measurements both at baseline and at follow-up were included for the validation analysis, which sought to demonstrate the validity of the predictions of the growth curves.

### 2.2. Statistical Analysis

All statistical analyses were performed using STATA version 17.0 (Stata Corp., stata.com). From the baseline cross-sectional data of the three grades (grades 1, 4, and 6), coefficients for AL and AL/CR ratio were determined using simultaneous quantile regression. The cut-off points were calculated for the 2nd, 5th, 10th, 25th, 50th, 75th, 90th, 95th, 98th, and 99th percentiles. These percentiles were chosen to enable comparison with the existing percentile charts of both European and Chinese children [6,7].

The ocular growth curve charts for AL and AL/CR ratio were generated using logistic quantile regression for the three grades, stratified by sex. Linear regression analysis was performed on the baseline data, with age, sex, and baseline AL and AL/CR as independent variables to predict follow-up AL and AL/CR ratios. Sensitivity and specificity parameters were measured to assess the usefulness of the cross-sectional percentile cut-offs in predicting myopia development among children, and a Receiver Operating Characteristic (ROC) curve analysis was performed where applicable. The data are presented as means (95% CI).

## 3. Results

The total number of children in the three grades at baseline was 4514. Boys represented 54% (n = 2442) of the overall sample. The mean age of the children in each grade was 6 (SD 0.48) years, 9 (SD 0.52) years, and 11 (SD 0.46) years, respectively. The ages of the children ranged from 5 to 12 years old. The prevalence of myopia at baseline was 11.7% (95% CI from 10.8 to 12.7%) in these three grades (grade 1: 10%; grade 4: 9.5%; grade 6: 15%). The mean spherical equivalent (SE) refraction was −0.01 (0.86) D. The refraction ranged from −8.10 D to +3.93 D in this cohort.

### 3.1. Axial Length Growth Curves and Percentiles

**Figure 1:**
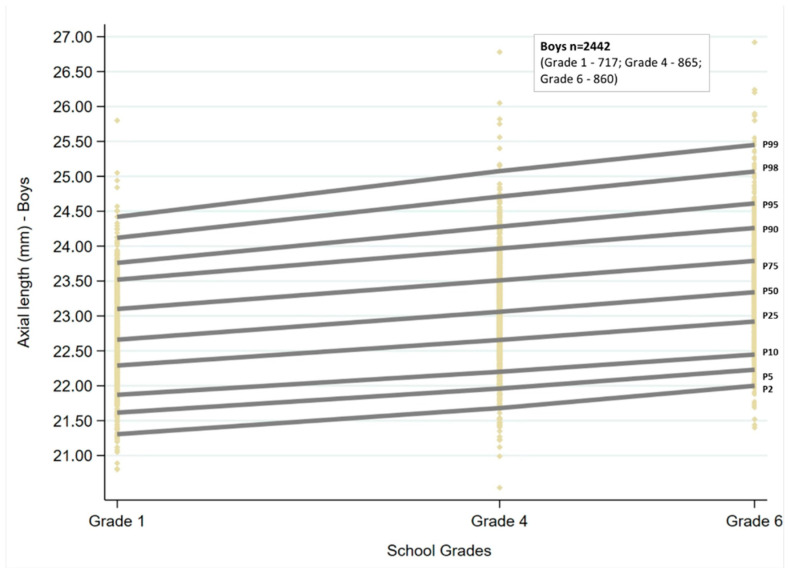
Axial length growth curves for boys from grades one, four, and six in the study sample. P2 to P99 represent the 2nd to 99th percentile. Each of the yellow diamonds represents a child in each of the grades.

**Figure 2:**
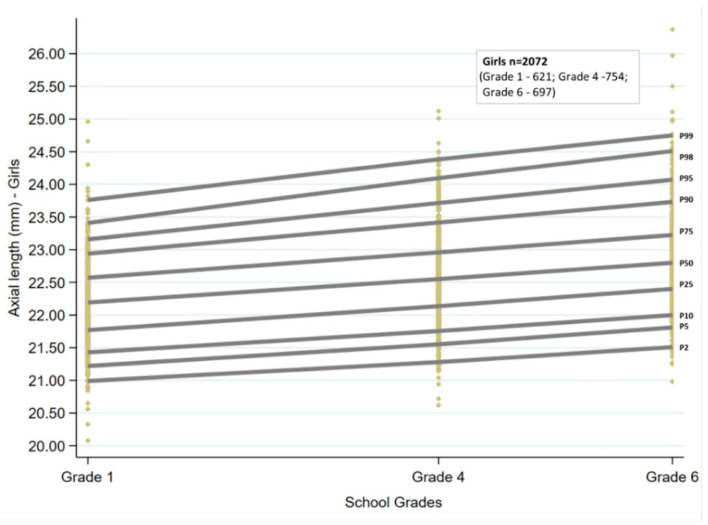
Axial length growth curves for girls from grades one, four, and six in the study sample. P2 to P99 represent the 2nd to 99th percentile. Each of the yellow diamonds represents a child in each of the grades.

Ocular growth curves for boys and girls for the three age groups are shown in Figure 1 and Figure 2. The corresponding percentile values are given in Table 1. Overall, for the age groups of children examined, the growth curves showed a linear trend. There was an increase in AL with increasing grade for both sexes, which was statistically significant for all the percentiles (*p* < 0.001). The AL increased by 0.08 to 0.09 mm for every unit increase in grade at the 25th, 50th, and 75th percentiles, and the change was similar for boys and girls. There was an increase of more than 0.1mm at the lowest and highest percentiles for both sexes (2nd, 5th, 98th, and 99th percentiles).

The AL cut-offs obtained by quantile regression for each of the grades stratified by sex are given in Table 1.

### 3.2. Axial Length to Corneal Curvature (AL/CR) Ratio Curves and Percentiles

**Figure 3:**
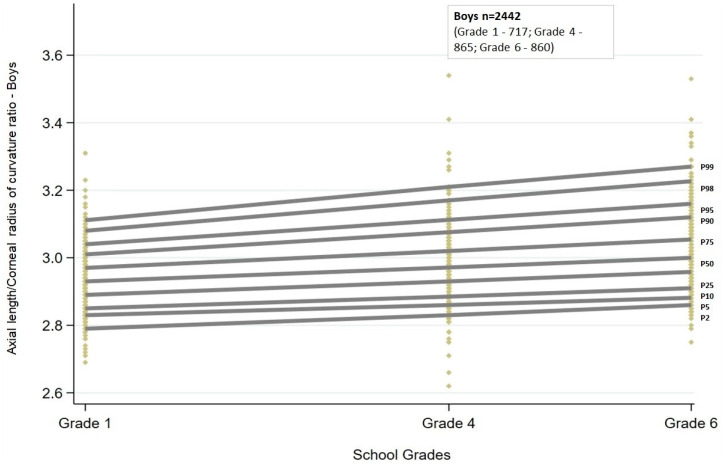
Percentile curves of axial length to corneal curvature ratio for boys from grades one, four, and six in the study sample. P2 to P99 represent the 2nd to 99th percentile. Each of the yellow diamonds represents a child in each of the grades.

**Figure 4:**
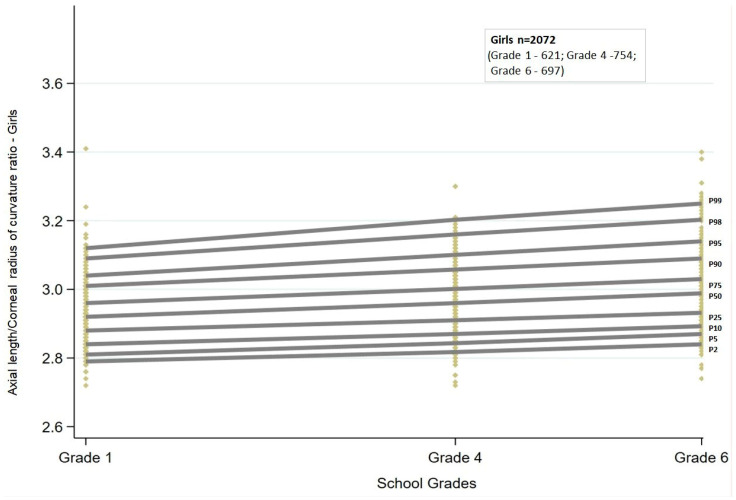
Percentile curves of axial length to corneal curvature ratio for girls from grades one, four, and six in the study sample. P2 to P99 represent the 2nd to 99th percentile. Each of the yellow diamonds represents a child in each of the grades.

Similar to AL, the AL/CR ratio also showed a linear trend. There was a statistically significant (*p* < 0.001) increase in the AL/CR ratio across all the percentiles. The increasing trend was similar between boys and girls (Figure 3 and Figure 4).

Among boys, the increase in AL/CR ratio with grade ranged between 0.06 and 0.07 from the 5th to 75th percentiles, and for the highest percentiles, the change was greater than 0.1 (90th and 95th percentiles: 0.10; 98th percentile: 0.13; 99th percentile: 0.15). In contrast, among girls, the change in AL/CR ratio was greater than 0.1 both at the lowest (2nd and 5th percentiles: 0.12) and at the highest percentiles (95th percentile: 0.12; 98th percentile: 0.14; 99th percentile: 0.17). The change in ratio was between 0.07 and 0.09 for percentiles between the 10th and 90th percentiles.

The AL/CR ratio cut-offs obtained by quantile regression for each of the grades stratified by sex are given in Table 2.

### 3.3. Validation of the Growth Curves

Validation of the percentile curves in predicting the onset of myopia among children was undertaken using the follow-up data of 414 children. These children had a complete set of ocular measurements, including refraction and biometry measurements, at both the baseline visit and at follow-up. Follow-up time ranged from 33 to 36 months. Among the 414 children, 37 had myopia at baseline, and data from these children were excluded from the analysis. The final sample consisted of 377 children, of whom 34 had incident myopia (≤−0.75 D) present by the follow-up visit, accounting for a 9% incidence over a three-year period. Among the new myopes, there were 3 in grade 1, 11 in grade 4, and 20 in grade 6 from the initial visit.

### 3.4. Validity of Using Axial Length and Axial Length to the Corneal Radius of Curvature Ratio to Predict Myopia Onset

#### 3.4.1. Axial Length

**Figure 5:**
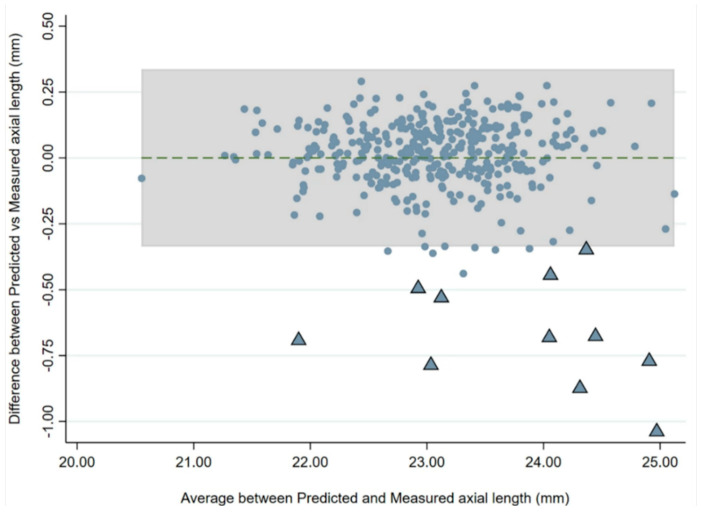
Bland–Altman plot showing the predicted axial length and the actual measured axial length at follow-up. The dotted line represents the mean difference, and the shaded portion represents the limits of agreement. The triangles represent the children with axial length changes outside the limits who became newly myopic by follow-up (11/17).

There was a strong correlation between baseline and follow-up AL and the AL/CR ratio. Linear regression analysis with baseline AL, sex, and age as dependent variables showed an R^2^ of 0.944 to predict AL at follow-up. A Bland–Altman analysis was used to assess agreement between AL values predicted from the regression and the actual AL values (Figure 5), and this revealed that 17 children (4.5%) had an AL change greater than predicted values. Eleven of these 17 children (65%) developed myopia [mean SE: −2.07 D (range: −1.12 D to −3.13 D)].

#### 3.4.2. Axial Length to Corneal Radius of Curvature Ratio

**Figure 6:**
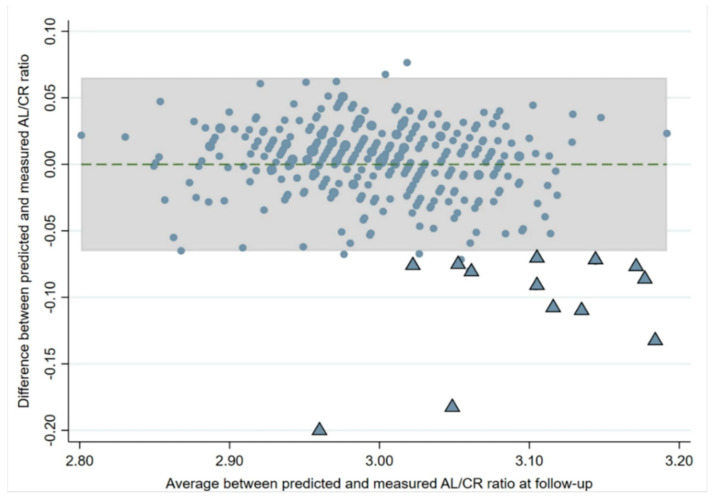
Bland–Altman plot between the predicted axial length to corneal curvature (AL/CR) ratio and the actual measured AL/CR ratio at follow-up. The dotted line represents the mean difference, and the shaded portion represents the limits of agreement. The triangles represent the children with AL/CR ratios outside the limits who became newly myopic by follow-up (14/20).

When regression analysis was used to predict the change in the AL/CR ratio at the follow-up visit from the baseline visit, the model had an R^2^ of 0.779 (*p* < 0.001) after adjusting for age at baseline and sex. A Bland–Altman plot (Figure 6) constructed to assess the agreement between the actual AL/CR ratio and the predicted AL/CR from the regression model showed that 20 children (5%) had a higher than predicted AL/CR ratio and were outside the limits of agreement. Fourteen of these children (70%) outside the limits of agreement had developed myopia by the follow-up visit (mean SE: −1.74 D; range: −0.75 D to −3.13 D).

### 3.5. Grade-Wise Axial Length, AL/CR Ratio Percentile, and Myopia Development

Based on the percentile values of AL and AL/CR ratio at baseline, each child was categorized as having myopia either absent or present (coded as 0 and 1) for the cut-offs given in Table 1 and Table 2, separated according to baseline grade and sex. The children in each grade were divided based on the 25th, 50th, 75th, and 90th percentile values. Since there were only three grade 1 children who developed myopia by the follow-up, sensitivity and specificity were not calculated for this group of children.

For the other two age groups, AL/CR percentiles were better for predicting myopia onset compared with AL alone. For the grade 4 children (mean age of 9 at baseline, n = 147), the AL/CR ratio at the 75th percentile had an overall sensitivity and specificity of 72.70% (95% CI 65.53–79.93%) and 85.29% (95% CI 79.57–91.02%), respectively, in predicting the development of myopia by grade 6 to 7 (mean age of 11). The area under the ROC (AUROC) for this cut-off was 0.79.

In contrast, for the 75th percentile of the AL cut-off, the sensitivity was 45.45% (95% CI 37.41–53.50%) and the specificity was 74.26% (95% CI 67.20–81.33%), respectively.

At grade 6 (mean baseline age 11, n =143), the 50th percentile of the AL/CR ratio had a sensitivity of 75.00% (95% CI 67.90–82.1%) and specificity of 69.92% (95% CI 62.40–77.44%) with an AUROC of 0.72 for predicting the probability of myopia development by grade 8 to 9 (mean age 13).

In comparison, for AL, the sensitivity and specificity for the 50th percentile in detecting myopia at higher grades were 55.00% (95% CI 46.85%–63.15%) and 56.91% (95% CI 48.79–65.03%), respectively.

### 3.6. Shift in Percentile Position and Myopia Development

When including the shift in AL percentile position from baseline to subsequent visit (centile crossing) as a predictor of myopia development along with baseline AL, the predictivity of the model improved with an AUC of 0.83 (sensitivity 90.90%; specificity 30.88%). Out of the 147 children in grade 4 who had information on centiles for two visits, 11 children were identified as newly myopic. Six out of these eleven children demonstrated centile crossing compared with baseline, and among the five children who did not show centile crossing, four were already in percentiles higher than the 75th at baseline.

In addition, irrespective of age and sex, an AL or AL/CR ratio threshold value of 23.30 (AUC 0.65) or 3.00 (AUC 0.83), respectively, was the optimal cut-off to best identify children with a risk of myopia development in this cohort.

## 4. Discussion

The results of the present study have provided percentile values for AL and the AL/CR ratio for South Indian children between the ages of 6 and 12. The percentile charts generated will aid in clinical monitoring of children and help in monitoring the response to myopia control treatments. Based on the results of this study, children with an ocular biometry profile (AL, AL/CR) falling on or above the 75th percentile at the age of 9 or the 50th percentile at age 11 are at risk of becoming myopic. These children should be offered intervention with myopia prevention strategies and closely monitored in a clinical or screening setting. Similarly, if a child’s axial length and AL/CR ratio are above the identified threshold (AL ≥ 23.30 mm and/or AL/CR ratio of 3.00), they have a higher risk of myopia development in follow-up. Children who show centile crossing compared with their initial visit should also be closely monitored. The usefulness of centile values and centile crossing in monitoring myopia development is similar to those seen in European and Chinese population studies [9,10,11,12,13,21].

However, when comparing with other ethnicities, Indian children had a shorter axial length in comparison to Chinese children of similar age for the 25th, 50th, and 75th percentiles at both 6 and 9 years of age [10]. The difference progressively increased from 0.32–0.36 mm at the age of 6, up to 1.14–1.27 mm at the age of 9 [10]. Similarly, when comparing the AL percentiles at 11 years of age with those from the study of He et al. [11], Indian children had a shorter AL across all percentiles when compared with Chinese children, irrespective of sex, though the difference was slightly less than that seen at the age of 9 years (mean difference: 0.87 mm for boys and 0.96 mm for girls).

In contrast, at the age of 6 years, a longer AL was noted among Indian children when compared to European children (mean difference 0.08 mm for boys and 0.12 mm for girls) [9] but a relatively shorter AL at age 9 years (mean difference 0.26 mm for boys and 0.21 mm for girls for the 50th percentile). Across all the percentiles, boys had a longer AL compared with girls, which was again consistent with other studies that have given percentile distributions of AL [9,10,11,12].

Similar to the AL percentiles, the AL/CR ratio percentiles also showed a linear trend, with an increase in ratio with increasing grade. Unlike AL, the AL/CR ratio did not differ greatly between boys and girls. When comparing by grade, the mean difference in the AL/CR ratio was 0.01 for the median percentile at both grades 1 and 4, whereas at grade 6, this difference slightly increased to 0.02. Overall, the change in AL/CR ratio from grade 1 to 6 was 0.07/0.06 (boys/girls) for the 50th percentile, and this difference increased to 0.15/0.14 (boys/girls) at the highest percentile (99th).

When comparing the AL/CR ratio of the present study with a previous study among Chinese children [11], at the age of 6 years, the AL/CR ratio was similar between the two populations, whereas with an increase in age, the ratios for the Chinese children were increasingly larger than for the Indian children across the percentiles. The median percentile at age 6 years was slightly larger for Indian children (mean difference 0.02 for boys and 0.03 for girls), whereas at age 11 years, children from China had a larger AL/CR ratio when compared with the present study group (mean difference 0.06 for boys and 0.07 for girls).

From the comparisons above, it can be noted that there is a minimal difference between different ethnicities at younger ages, whereas for the older age groups, the cut-offs vary with longer growth and change noted for other ethnicities, possibly due to both genetic and lifestyle differences between the populations. This also translated into myopia-prevalence differences being observed between ethnicities. Both Chinese and European children had a similar myopia prevalence at the age of 5 years (4 to 6%) [9,11]; however, prevalence reached 90% for Chinese children by 16 years, whereas for the European cohort, the prevalence was less (around 28%) by age 18 [11]. The prevalence of myopia in the STEM cohort was close to 10% until the age of 10 years and increased to 24% by age 15 [1].

When analyzing the usefulness of ocular biometry measures to predict myopia, in the validation sample, baseline AL predicted the change in AL in the follow-up with good agreement. Of the 377 children, 4% (n = 17) had an axial length change that was outside the limits of agreement between the actual and predicted change (>0.3 mm in three years). When analyzing the children whose AL was outside the predicted growth range, 11 children had developed myopia by the follow-up visit. Our results are comparable with previous studies that found a rapid increase in AL prior to the onset of myopia [22]. While we do not know the eventual refractive fate of the six children outside the limits of agreement who had not yet developed myopia, they may represent a pre-myopic or still at-risk cohort.

The sensitivity analysis showed that AL alone may not be the optimum predictor to identify myopia onset, and one possible reason could be that the number of children who became newly myopic was relatively small in the follow-up cohort (9% over a three-year period), and a longer follow-up may be needed to further verify the finding. In addition to an AL cut-off >23.07 mm, adding centile crossing (moving up one centile group/ band or more) in the percentile chart in a subsequent follow-up visit as another predictor improved myopia prediction accuracy (sensitivity and specificity—89% and 69%, respectively) in a study among European children [21]. In the present study, similar findings were noted. Six children in grade 4 who were newly myopic had moved up at least one centile by grade 6. Shifting between centile curves could therefore be useful to identify children who are at risk of developing myopia. Further validation, preferably with a longer follow-up duration, can confirm the findings and further strengthen the growth percentiles. As our study population is limited to a younger age group, the usefulness of AL growth charts in monitoring myopia progression can be validated through future follow-up of this cohort from the STEM study and analysis of their second set of follow-up data.

Our data indicated that the AL/CR ratio is a more appropriate predictor for myopia development [23,24]. In the sensitivity analysis, the AL/CR ratio in the 75th percentile (at age 9 years) had a sensitivity of 72.70% and a specificity of 85.29% in predicting myopia development by age 11 years. Similarly, an AL/CR ratio in the 50th percentile at age 11 had a 75.00% sensitivity and specificity of 69.92% in predicting myopia by the age of 13 years. Both AL and AL/CR ratio percentiles can be used as screening tools to identify children who are at risk of developing myopia in conjunction with other risk factors.

There are a few limitations to the present study. The study used open-field, non-cycloplegic autorefraction instead of the gold standard, cycloplegic refraction, representing a potential limitation of the study. This being said, this methodological approach has previously been validated for reliable refraction measurements in concert with a slightly modified definition of myopia (spherical equivalent refraction (SE) of ≤−0.75 D) instead of the standard definition of SE ≤ −0.50 D [18]. In addition, the study was primarily conducted in the southern state of India and comprised predominantly urban and suburban regions. Thus, the findings may not be generalizable to other regions of the country. It follows that the developed ocular growth curve charts may benefit from further strengthening with similar data collected across other regions of India, including rural regions where the reported myopia prevalence is low [25]. Also, the present study provides data from three age groups, and there is a need to develop similar data for other age groups, including adults, to better understand myopia progression. Similarly, growth curves to understand normal emmetropic eye growth could further help in understanding the efficacy of myopia control treatments.

In conclusion, while evaluating Indian children who are at risk of myopia onset, close monitoring and early intervention could be applied to children who are at a higher percentile than the median (>50th) and/or children with an axial length of 23.30 mm or an AL/CR ratio of 3 or greater. Similarly, a shift in centile at follow-up visits could indicate possible myopia development, and frequent follow-ups and timely intervention might be offered to such children.

## Figures and Tables

**Table 1:** Axial length cut-offs for the various percentiles with the upper and lower 95% confidence intervals for boys and girls across the three grades in the baseline study sample.

Percentiles	Axial Length Cut-Offs-Boys (mm)			Axial Length Cut-Offs-Girls (mm)		
	Grade 1 (n = 717)	Grade 4 (n = 865)	Grade 6 (n = 860)	Grade 1 (n = 621)	Grade 4 (n = 754)	Grade 6 (n = 697)
2	21.37 (21.23–21.51)	21.54 (21.39–21.69)	22.01 (21.94–22.08)	20.99 (20.87–21.11)	21.27 (21.17–21.37)	21.51 (21.40–21.62)
5	21.61 (21.51–21.71)	21.97 (21.91–22.03)	22.23 (22.13–22.33)	21.22 (21.11–21.33)	21.57 (21.44–21.70)	21.80 (21.71–21.90)
10	21.88 (21.74–22.02)	22.17 (22.09–22.25)	22.48 (22.40–22.56)	21.40 (21.31–21.49)	21.83 (21.75–21.91)	21.97 (21.91–22.03)
25	22.29 (22.24–22.34)	22.65 (22.59–22.71)	22.92 (22.86–22.98)	21.76 (21.70–21.82)	22.17 (22.08–22.26)	22.37 (22.32–22.42)
50	22.67 (22.61–22.73)	23.05 (22.98–23.12)	23.35 (23.28–23.42)	22.18 (22.11–22.25)	22.58 (22.56–22.63)	22.79 (22.73–22.85)
75	23.11 (23.04–23.18)	23.50 (23.42–23.58)	23.79 (23.73–23.85)	22.54 (22.47–22.61)	22.99 (22.92–23.06)	23.19 (23.11–23.27)
90	23.55 (23.44–23.66)	23.92 (23.84–24.00)	24.29 (24.16–24.42)	22.93 (22.84–23.02)	23.42 (23.36–23.48)	23.73 (23.59–23.87)
95	23.79 (23.64–23.94	24.23 (24.09–24.37)	24.65 (24.52–24.78)	23.16 (23.04–23.28)	23.73 (23.62–23.84)	24.04 (23.84–24.24)
98	24.12 (23.86–24.38)	24.66 (24.44–24.88)	25.11 (24.85–25.37)	23.41 (23.18–23.64)	24.07 (23.90–24.24)	24.51 (24.20–24.82)
99	24.42 (23.97–24.87)	25.08 (24.61–25.55)	25.45 (25.00–25.90)	23.76 (23.50–24.02)	24.34 (24.15–24.53)	24.77 (24.44–25.10)

**Table 2:** Axial length to corneal curvature ratio cut-offs for the various percentiles with the upper and lower 95% confidence intervals for boys and girls across the three grades in the study sample.

Percentiles	AL/CR Ratio-Boys			AL/CR Ratio-Girls		
	Grade 1 (n = 717)	Grade 4 (n = 865)	Grade 6 (n = 860)	Grade 1 (n = 621)	Grade 4 (n = 754)	Grade 6 (n = 697)
2	2.79 (2.77–2.81)	2.82 (2.81–2.83)	2.86 (2.85–2.87)	2.79 (2.77–2.80)	2.82 (2.81–2.83)	2.83 (2.82–2.84)
5	2.83 (2.82–2.84)	2.86 (2.85–2.87)	2.89 (2.88–2.90)	2.82 (2.81–2.83)	2.84 (2.83–2.85)	2.87 (2.85–2.89)
10	2.85 (2.84–2.86)	2.89 (2.88–2.90)	2.91 (2.90–2.92)	2.84 (2.83–2.85)	2.87 (2.86–2.88)	2.90 (2.89–2.91)
25	2.89 (2.88–2.90)	2.93 (2.925–2.93)	2.95 (2.94–2.96)	2.88 (2.87–2.89)	2.91 (2.90–2.92)	2.93 (2.92–2.94)
50	2.93 (2.92–2.94)	2.97 (2.965–2.97)	3.00 (2.99–3.01)	2.92 (2.91–2.93)	2.96 (2.955–2.964)	2.98 (2.97–2.99)
75	2.97 (2.96–2.98)	3.02 (3.01–3.03)	3.06 (3.05–3.07)	2.96 (2.95–2.97)	3.00 (2.99–3.01)	3.03 (3.02–3.04)
90	3.02 (3.01–3.03)	3.07 (3.06–3.08)	3.12 (3.11–3.13)	3.01 (3.00–3.02)	3.05 (3.04–3.06)	3.09 (3.07–3.11)
95	3.04 (3.03–3.05)	3.10 (3.08–3.12)	3.16 (3.14–3.18)	3.04 (3.02–3.06)	3.11 (3.09–3.13)	3.14 (3.12–3.16)
98	3.08 (3.05–3.11)	3.17 (3.13–3.21)	3.22 (3.19–3.25)	3.10 (3.07–3.13)	3.15 (3.13–3.17)	3.22 (3.17–3.27)
99	3.12 (3.06–3.18)	3.21 (3.15–3.27)	3.27 (3.22–3.32)	3.12 (3.08–3.16)	3.17 (3.14–3.20)	3.26 (3.23–3.29)

## Data Availability

The original contributions presented in the study are included in the article, further inquiries can be directed to the corresponding author.
